# Organ doses evaluation for chest computed tomography procedures with TL dosimeters: Comparison with Monte Carlo simulations

**DOI:** 10.1002/acm2.12505

**Published:** 2018-12-03

**Authors:** Louise Giansante, Juliana C. Martins, Denise Y. Nersissian, Karen C. Kiers, Fernando U. Kay, Marcio V. Y. Sawamura, Choonsik Lee, Eloisa M. M. S. Gebrim, Paulo R. Costa

**Affiliations:** ^1^ Group of Radiation Dosimetry and Medical Physics Institute of Physics University of São Paulo (IFUSP) São Paulo SP Brazil; ^2^ Ludwig‐Maximilians‐Universität München (LMU) Munich Germany; ^3^ Vrije Universiteit Amsterdam (VU) Amsterdam The Netherlands; ^4^ Institute of Radiology School of Medicine University of São Paulo (InRad) São Paulo SP Brazil; ^5^ Division of Cancer Epidemiology & Genetics National Cancer Institute National Institutes of Health (NIH) Bethesda MD USA

**Keywords:** computed tomography, dosimetry/exposure assessment, image quality, Monte Carlo simulations, organ dose

## Abstract

**Purpose:**

To evaluate organ doses in routine and low‐dose chest computed tomography (CT) protocols using an experimental methodology. To compare experimental results with results obtained by the National Cancer Institute dosimetry system for CT (NCICT) organ dose calculator. To address the differences on organ dose measurements using tube current modulation (TCM) and fixed tube current protocols.

**Methods:**

An experimental approach to evaluate organ doses in pediatric and adult anthropomorphic phantoms using thermoluminescent dosimeters (TLDs) was employed in this study. Several analyses were performed in order to establish the best way to achieve the main results in this investigation. The protocols used in this study were selected after an analysis of patient data collected from the Institute of Radiology of the School of Medicine of the University of São Paulo (InRad). The image quality was evaluated by a radiologist from this institution. Six chest adult protocols and four chest pediatric protocols were evaluated. Lung doses were evaluated for the adult phantom and lung and thyroid doses were evaluated for the pediatric phantom. The irradiations were performed using both a GE and a Philips CT scanner. Finally, organ doses measured with dosimeters were compared with Monte Carlo simulations performed with NCICT.

**Results:**

After analyzing the data collected from all CT examinations performed during a period of 3 yr, the authors identified that adult and pediatric chest CT are among the most applied protocol in patients in that clinical institution, demonstrating the relevance on evaluating organ doses due to these examinations. With regards to the scan parameters adopted, the authors identified that using 80 kV instead of 120 kV for a pediatric chest routine CT, with TCM in both situations, can lead up to a 28.7% decrease on the absorbed dose. Moreover, in comparison to the standard adult protocol, which is performed with fixed mAs, TCM, and ultra low‐dose protocols resulted in dose reductions of up to 35.0% and 90.0%, respectively. Finally, the percent differences found between experimental and Monte Carlo simulated organ doses were within a 20% interval.

**Conclusions:**

The results obtained in this study measured the impact on the absorbed dose in routine chest CT by changing several scan parameters while the image quality could be potentially preserved.

## 1 INTRODUCTION

X‐ray computed tomography (CT) became clinically available in the beginning of the 1970s, innovating the practice of Medicine by substantially decreasing the need of exploratory surgery.^1^ Since the development of the first CT equipment, this diagnostic imaging modality has been rapidly expanding, mainly due to the speed of image acquisition, and high‐quality images.^2^ Surveys such as the conducted in the United States in 1987 estimated that in 1980, only few years after its implementation, 2.2 million CT procedures were performed in general hospitals.^3^ In 2007, it was estimated that more than 62 million CT procedures had been performed, from which at least 4 million were pediatric examinations.^4^ Chest CT is one of the most common imaging examinations performed, accounting for approximately 16% of all CT procedures.^5^ Notwithstanding, its utilization is increasing due to relatively recent efforts to implement low‐dose chest CT for lung cancer screening in high‐risk populations. As a consequence of the increasing number of CT examinations, the radiation dose absorbed by patients has become a concern among radiologists, researchers, and manufacturers.^4^, ^6^ Currently, CT utilization faces challenges related to justification of the procedure (i.e., benefits should outweigh potential risks) and dose optimization.^7^, ^8^

With the development of the CT technology, scanners have become more complex and efficient, challenging the accuracy of traditional dosimetry methods.^1^ Although the computed tomography dose index (CTDI) and the dose length product (DLP) are well stablished metrics nowadays, these quantities only provide the information about how the machine was operated.^9^ However, much more important and complex to assess is the information on the patient dose from any arbitrary examination. This information depends on a number of parameters, such as patient size and the anatomical region scanned.^10^

Efforts have been made to develop robust methodologies to allow direct estimation of organ doses from patients undergoing CT exams. New ancillary metrics for CT dose quantification are being developed, such as the effective diameter and water‐equivalent diameter, which are adopted to assess the size specific dose estimates (SSDE).^11^, ^12^ The correlation between the aforementioned quantity and organ doses is still under investigation.^13^

Estimation of organ dose values is not a trivial task. In general, three approaches have been adopted over the past decades: (a) direct measurements with different kinds of dosimeters, anthropomorphic phantoms, and postmortem subjects, (b) calculations using Monte Carlo methods combined with computational human phantoms, and (c) biological dosimetry based on blood samples.^10^ Several advantages and disadvantages can be discussed regarding each approach. Anthropomorphic phantoms for dosimetry, for instance, have been in use for more than 30 yr, and researches indicate the ongoing development of phantoms according to new CT technologies.^14^ The use of *postmortem* subjects provides a wide range of different sizes and anatomies. However, they do not replace the use of phantoms. This technique is difficult to perform and dose measurement is limited to some points, thus it is difficult to measure the average dose to a given organ.^10^ Monte Carlo simulations generate accurate 3D dose distributions while it is less time‐consuming and more flexible. On the other hand, the increasing use of proprietary scanning techniques by CT vendors adds a difficulty on the accurate implementation in Monte Carlo simulations, which is not an issue for direct experimental measurements.^15^ Biological dosimetry, based on analyzing patient's blood before and after a CT scanner to evaluate the DNA's damage caused by the exposure to X ray, is time‐consuming, costly, and does not provide an evaluation of dose to individual organs.^10^ Considering these advantages and disadvantages and taking into account their previous experience on TL dosimetry and Monte Carlo simulations, the authors elected the present approach, which compares organ dose results estimated from both methods.

In this study, an experimental methodology to evaluate organ doses in routine and low‐dose chest CT protocols was the approach of choice. This method consists of using Lithium Fluoride doped with Magnesium and Titanium (LiF:Mg,Ti) thermoluminescent dosimeters (TLDs) chips embedded in adult and pediatric anthropomorphic phantoms. Besides the advantages of using anthropomorphic phantoms previously pointed, Lithium Fluoride TLD dosimeters are tissue‐equivalent, thus it is not necessary to correct for the energy dependence in the energy range of radiology and radiotherapy.^16^ Moreover, their small sizes provide accurate spatial localization of the doses inside the studied organs. These measurements were compared with dose estimates obtained with Monte Carlo simulations using National Cancer Institute dosimetry system for CT (NCICT^a^), an organ dose calculator based on Monte Carlo radiation transport technique combined with a series of computational human phantoms.^17^ In addition, this investigation also addresses the effects of tube current modulation (TCM) on organ dose in comparison with fixed tube current protocols, particularly in pediatric examinations in which TCM protocols have been recently applied for chest CT irradiations. However, its efficiency has been questioned for pediatric patient irradiations.^18^, ^19^ As the standard protocol for pediatric chest CT in InRad involves TCM, the effects on TCM on organ dose in comparison to protocols with fixed tube current were evaluated.

## 2 MATERIALS AND METHODS

### 2.A Thermoluminescent dosimeters

Lithium Fluoride doped with Magnesium and Titanium (LiF:Mg,Ti) thermoluminescent dosimeters (TLD), in the format of 3 × 3 × 1 mm^3^ chips (TLD‐100, Harshaw Chemical Company, OH, USA) were used in the present work. These TLD chips were processed by a Risφ TL/OSL reader, model DA‐20, (DTU Nutech. Inc., Roskilde, Denmark). During the reading process, the dosimeters were heated from room temperature to 350°C at a constant rate of 10°C/s, generating the LiF:Mg,Ti characteristic TL curve (photon counts against temperature). The so‐called “TL value” was then obtained by numerically integrating the TL curve and the resulting quantity is directly proportional to the dose deposited by the radiation in the dosimeter.^20^

In order to correlate the TL value to the Air Kerma (*K*_Air_), calibration curves were constructed using both an RQT 9 X ray beam quality^21^ generated by a Philips MCN 421 equipment (Philips, Germany) and a Philips Brilliance 64 CT scanner.^22^ Two SSDL calibrated ion chambers (30 cc from PTW, Freiburg, Germany, and 0.6 cc from Radcal Corporation, Monrovia, CA, USA) were used to measure the air kerma. These calibration curves were adopted for the organ doses estimations with the anthropomorphic phantoms.

### 2.B Anthropomorphic phantoms

Two anthropomorphic phantoms were used in this study. A RANDO Phantom (The Phantom Laboratory, Salem, NY, USA) simulates the anatomical characteristics of the Reference Man^23^ and it consists of a real human skeleton embedded in soft tissue‐equivalent material.^24^ The other phantom adopted was the CIRS ATOM^®^ dosimetry verification phantom, model 705 (CIRS, Inc., Norfolk, VA, USA), which simulates a pediatric 5‐yr‐old patient. In every slice of both phantoms, drilled holes enable the introduction of different types of dosimeters.

**Figure 1:**
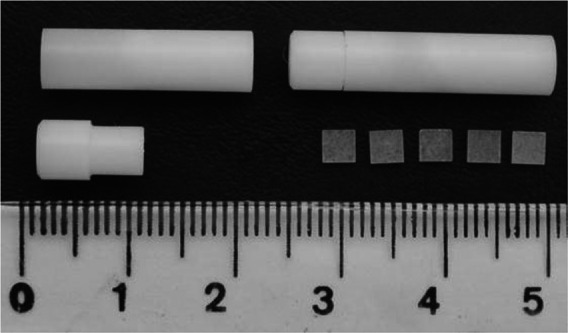
Thermoluminescent dosimeter holder, specially designed to be introduced into RANDO phantom internal holes, and the TLD chips placed beside a scale for perspective view.

Dosimeter holders were specially designed using polyoximethylene to accommodate up to 5 TLDs inside the drilled holes of the anthropomorphic phantoms.^25^ Figure 1 shows two dosimeter holders together with TLDs and a centimeter scale for perspective.

### 2.C CT scanners

The irradiations were performed using two different 64‐slice CT scanners from the Institute of Radiology of the School of Medicine of the University of São Paulo. For all the chest protocols, the adult phantom was irradiated in a GE Discovery CT 750 HD (GE Healthcare, Waukesha, WI, USA), whereas a Philips Brilliance 64 CT scanner (Philips Healthcare, Bothell, WA, USA) was used for irradiations of the pediatric phantom, as pediatric examinations were mostly performed in this equipment.

### 2.D CT acquisition protocols

#### 2.D.1 Adult protocols

Data from the picture archiving and communication system/radiology information systems (PACS/RIS) from the Institute of Radiology of the School of Medicine of the University of São Paulo were analyzed regarding the variety and frequency of CT protocols. The results of this frequency study were used for choosing the most relevant CT procedures that totally irradiated chest region and showed to be relevant for lung dose evaluation. Three different chest protocols with constant tube current were identified during the PACS survey, here denominated “Standard” (STD), “Low Dose” (LD), and “Ultra Low Dose” (ULD). The STD protocol is designed for detection and diagnosis of chest wall, pleural, pulmonary, and mediastinal disease. In contrast, LD and ULD are protocols optimized for detection of lung diseases. The LD protocol was designed according to guidelines for lung cancer screening (NCCN Clinical Practice Guidelines in Oncology, version 2.2016) recommending scanning parameters set at 100–120 kV and 40–60 mAs for a standard man.^26^ The ULD protocol was designed as part of an ongoing investigation approved by the institutional review board to address the diagnostic information of CT scans with doses comparable to chest radiographs.^27^ Other investigators have previously reported this practice for dose optimization.^28^, ^29^, ^30^ Both LD and ULD protocols seek to reduce the dose by adjusting the scanner's tube current. LD tube current is set at 120 mA, with 48 mAs, whereas ULD is set at an even lower value of 40 mA, with 16 mAs, which represents a significant decrease compared to the value of 300 mA used for STD chest CT protocol.

Two phantom irradiations were performed to investigate the impact of TCM on lung dose reduction in the GE scanner. One irradiation consisted of longitudinal TCM (“Auto mA”), whereas the second irradiation consisted of both longitudinal and angular modulation combined (“Auto + Smart mA”). The angular modulation in GE scanners can only be selected in combination with longitudinal modulation.^31^ Acquisition parameters of the studied protocols are presented in Table 1. Since scan projection radiographs (SPR) are often performed before TCM protocols, the imparted doses due to double SPRs were also evaluated.

**Table 1:** Acquisition parameters for the adult phantom irradiation using the GE CT scanner. The values for CTDI_vol_ and DLP displayed by the scanner, relative to a 32 cm CTDI phantom, are also shown

	SPR	Constant tube current			Tube current modulation	
		Standard	Low dose	Ultra low dose	Auto mA	Auto + Smart mA
Tube voltage (kV)	120					
Tube current (mA)	10	300	120	40	80–300	80–300
Rotation time (s)	–	0.6	0.4	0.4	0.6	0.6
Pitch	–	1.375	1.375	1.375	1.375	1.375
Collimation (mm)	–	64 × 0.625	64 × 0.625	64 × 0.625	64 × 0.625	64 × 0.625
CTDI_vol_ (mGy)	–	10.03	2.76	0.92	6.61	5.59
DLP (mGy cm)	–	465.90	128.52	42.81	306.96	259.49

#### 2.D.2 Pediatric protocols

Diagnostic pediatric chest CT were also surveyed using information obtained from the institutional PACS, using DICOM header metadata. The target protocol for this study was named “Chest for Children,” which is the standard chest protocol for pediatric population. In order to compare doses under different operating conditions, four variations of this protocol were assessed: two values of tube voltage were used (120 and 80 kV), and for each tube voltage, first a fixed mAs value was chosen and then longitudinal TCM was used. However, this approach differs from clinical practice, as TCM is always selected regardless of tube voltage for dose reduction. The acquisition parameters of the studied protocols are presented in Table 2.

**Table 2:** Acquisition parameters for the pediatric phantom irradiation using the Philips CT scanner. The values for CTDI_vol_ and DLP displayed by the scanner, relative to a 32 cm phantom, are also shown

	Chest for children			
	Constant tube current		Longitudinal tube current modulation	
Tube voltage (kV)	120	80	120	80
Tube current (mA)	121	323	66–118	168–318
Rotation time (s)	0.45	0.45	0.45	0.45
Pitch	0.922	0.922	0.922	0.922
Collimation (mm)	64 × 0.625	64 × 0.625	64 × 0.625	64 × 0.625
CTDI_vol_ (mGy)	3.7	2.8	2.8	2.2
DLP (mGy cm)	103.6	76.4	85.0	65.3

### 2.E TLD positioning

TLD groups were positioned inside the phantoms according to the thyroid and lung distributions^25^, ^32^, ^33^, ^34^ (Tables 3 and 4). In every irradiation, one group of TLD was left outside the examination room in order to estimate the background radiation dose, which was subtracted from all TL values corresponding to the irradiations during data analysis. The placement of the groups inside each phantom is described below.

**Table 3:** TLD groups distributed inside the RANDO Phantom for studied protocols and double SPR, and corresponding lung mass fraction. $f_{i}$ is the lung fraction contained in ith slices.^25^, ^33^, ^34^

Slice (i)	$f_{i}$ lung	Number of TLD groups
11	0.06	2
12	0.09	2
13	0.11	6
14	0.14	6
15	0.14	8
16	0.13	6
17	0.13	4
18	0.11	4
19	0.09	2

**Table 4:** TLD groups distributed inside the CIRS ATOM Phantom for all studied chest protocols, and corresponding organ mass fractions.^32^

Slice (*i*)	Organ	*f*_*i*_	Number of TLD groups
8	Thyroid	1.00	4
9	Lungs	0.02	2
10		0.14	4
11		0.19	6
12		0.22	6
13		0.23	8
14		0.17	4
15		0.03	2

#### 2.E.1 Adult phantom

All adult chest irradiations were performed using 40 groups of three TLDs each distributed into the lungs of the adult phantom. The distribution of the groups within each slice of the phantom along with the lung tissue fraction is presented in Table 3. In Table 3, *f*_*i*_ values correspond to the lung mass fraction contained inside each physical slice *i* of the phantom.

#### 2.E.2 Pediatric phantom

All pediatric chest irradiations were performed using 36 groups of three TLDs each, from which 32 were placed in the lungs and 4 were placed in the thyroid.

The distribution of the groups within each slice of the phantom along with the lung and thyroid tissue fraction is presented in Table 4. The determination of the fractions of the total lung mass (*f*_*i*_) is described elsewhere.^32^

In a typical chest CT procedure, the lungs are entirely irradiated and the thyroid is at least partially irradiated, according to the position of the patient on the couch. Since those are radiosensitive organs,^2^ it is important to evaluate the radiation dose absorbed by these organs during such procedures. Thyroid doses evaluation is particularly relevant for pediatric patients due to their long life expectancy. Therefore, the pediatric phantom was irradiated from the middle of the neck through the lung bases and the resulting doses to the lungs and to the thyroid were evaluated.

### 2.F Organ doses estimate

In order to convert the TL values into organ‐absorbed doses, the following 4‐step procedure was adopted:

- The TL values were converted into $K_{\mathrm{Air}}$, using the calibration curve previously described (Section 2.A).
- For each *phantom* slice i, a mean value of $K_{\mathrm{Air}}$ ($K_{\mathrm{Air}}^{i}$) is calculated, as shown in eq. (1).^25^, ^35^

(1) $$K_{\mathrm{Air}}^{i}=\frac{\sum_{n=1}^{G}\left(K_{\mathrm{Air}}^{n}/\sigma_{n}^{2}\right)}{\sum_{n=1}^{G}\left(1/\sigma_{n}^{2}\right)}.$$

where *G* is the total number of TLD groups accommodated into *i*th slice and $\sigma_{n}^{2}$ is the variance of the TL values from TLDs in the *n*th group. Equation (1) assumes purely statistical uncertainties from each TLD Group, since each group is not affected by partial volume irradiations, and it represents the weighted mean of individual air‐kerma means calculated from each TLD group inserted in the *i*th slice.^36^

- $K_{\mathrm{Air}}^{i}$ values were converted to organ average absorbed dose in the organ fraction present at *i*th slice, $D_{i}$, according to^25^, ^37^, ^38^:

(2) $$D_{i}=K_{\mathrm{Air}}^{i}\frac{{\left(\mu /\rho \right)}_{\mathrm{Organ}}}{{\left(\mu /\rho \right)}_{\mathrm{Air}}},$$

where (μ*/*ρ)_Organ_ and (μ/ρ)_Air_ are the mass‐energy absorption coefficients for the target organ and air^39^ respectively, which vary according to the effective energy of the X ray beam (Table 5). The determination of those values is described elsewhere.^25^, ^32^

- Last, the mean absorbed dose for the entire organ was estimated by summing up the contributions regarding each slice, where $f_{i}$ is the organ fraction contained in *i*th slice.^40^, ^41^

(3) $$D=\sum f_{i}\times D_{i}$$

**Table 5:** Mass‐energy absorption coefficients obtained for each compound and applied to estimate the organ doses

		**Lung tissue**	**Thyroid**	**Air (sea level)**
$\left(\frac{\mu_{\mathrm{en}}}{\rho }\right)\left(\frac{{\text{cm}}^{2}}{g}\right)$	120 kV	0.0365	0.0402	0.0339
	80 kV	0.0557	0.0610	0.0521

The uncertainties on organ dose values were considered within a 68.3% interval (*k* = 1) and are described in Appendix A.

### 2.G Comparison with NCICT

The results obtained with the experimental method proposed in this study were compared with the organ doses calculated by NCICT software. NCICT is based on a series of pediatric and adult computational human phantoms representing the reference individuals defined in the ICRP Publication 89 with several CT scanner models.^17^, ^42^, ^43^ The program features a graphical user interface so that the user can introduce the scan parameters specific to each examination.^17^ Moreover, the software comprises a batch module that enables the calculation of organ doses for a large number of patients and for a TCM protocol.^17^ The organ dose calculated from the software has been extensively tested by measurements.^44^, ^45^ Comparison results are presented along with the percent differences between experimental (*D*_exp_) and simulated (*D*_sim_) values per organ, as follows:

(4) $$\Delta =\left(\frac{D_{\mathrm{sim}}-D_{\exp}}{D_{\mathrm{sim}}}\right)\times 100\%$$

### 2.H Statistical evaluation

The agreement between experimental and simulated methods was quantified according to the Bland–Altman analysis.^46^ This analysis is used to evaluate the mean differences between two different methods by estimating an agreement interval, in which 95% of these differences fall.^46^, ^47^ In this study, the percent differences between experimental and simulated doses (*D*_exp_ and *D*_sim_, respectively) were plotted against their means ($\frac{D_{\mathrm{sim}}+D_{\exp}}{2}$) and the limits of agreement were determined using RStudio software (RStudio, Inc. Boston, MA, USA).

## 3 RESULTS

### 3.A CT acquisition protocols

**Figure 2:**
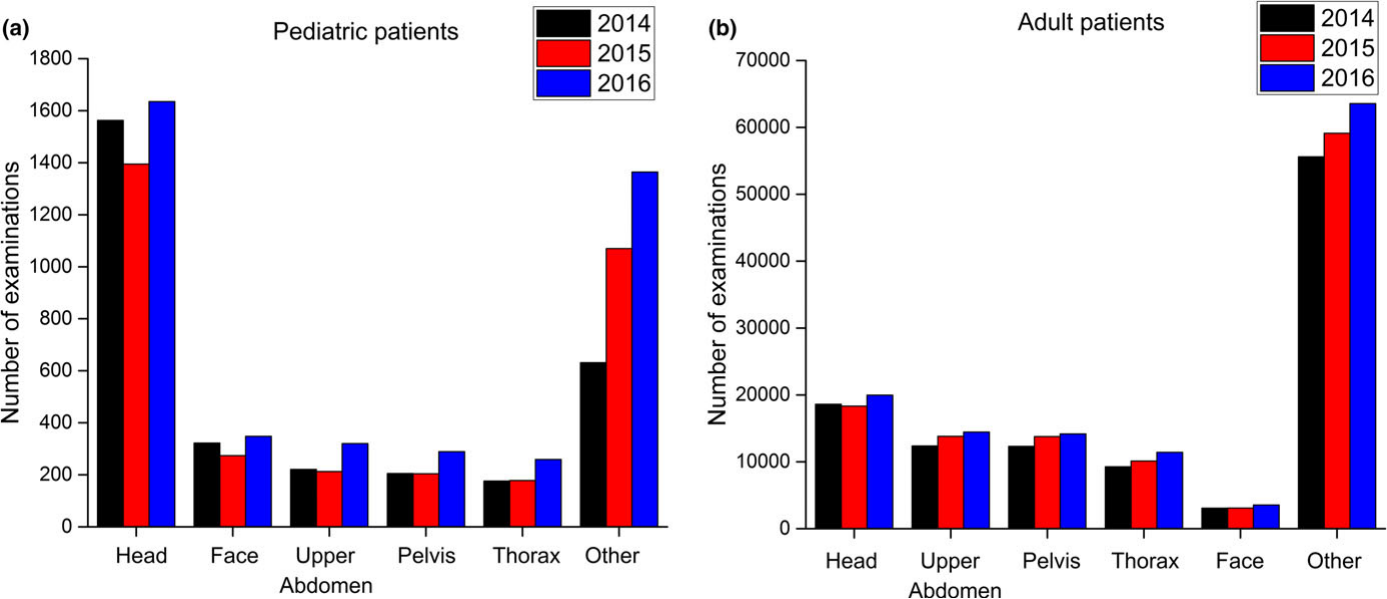
Five most applied CT protocols for pediatric (left) and adult (right) patients at InRad during the years 2014–2016. Chest CT is the 5th most applied protocol in pediatric patients and the 4th most applied protocol in adult patients.

The evaluation of the CT examinations conducted at InRad showed that more than 50 modalities of CT are performed annually. In 2016, a total of 95,000 patients were identified. About 5% of these patients were pediatric (0–15 yr old). The most frequently applied protocols for both adult and pediatric patients were identified (Fig. 2).

### 3.B Organ doses estimate

#### 3.B.1 Adult lung doses

The lung mean absorbed doses due to the Chest CT protocols previously described are summarized in Table 6, along with further dosimetric quantities (dose/mAs, dose/mAs_eff_, CTDI_vol_, and DLP values).

**Table 6:** Lung‐absorbed doses and further dosimetric quantities with respective uncertainties (*k* = 1) for the Chest protocols applied to the adult phantom

	SPR	Chest protocols — constant tube current			Chest protocols — tube current modulation/TCM	
		Standard	Low dose	Ultra low dose	Auto mA	Auto + Smart mA
Dose/mAs (mGy/mAs)	–	0.0794 ± 0.0009	0.081 ± 0.001	0.078 ± 0.001	–	–
Dose/mAseff (mGy/mAseff)	–	0.109 ± 0.001	0.111 ± 0.001	0.107 ± 0.001	–	–
Lung mean absorbed dose (mGy)	0.19 ± 0.01	14.30 ± 0.70	3.88 ± 0.19	1.24 ± 0.06	11.90 ± 0.60	9.29 ± 0.46

#### 3.B.2 Pediatric lung and thyroid doses

For the pediatric phantom, doses to the lungs and thyroid were evaluated. These organs were directly irradiated by the primary beam of the chest CT scan. Results are presented in Table 7, along with further dosimetric quantities (dose/mAs, dose/mAs_eff_, CTDI_vol_, and DLP values).

**Table 7:** Organ‐absorbed doses and further dosimetric quantities with respective uncertainties (*k* = 1) for the Chest protocols applied to the pediatric phantom

Chest for children protocol				
	Constant tube current		Longitudinal tube current modulation	
Thyroid				
Dose/mAs (mGy/mAs)	0.124 ± 0.004	0.041 ± 0.002	–	–
Dose/mAseff (mGy/mAseff)	0.115 ± 0.004	0.037 ± 0.002	–	–
Mean absorbed dose (mGy)	6.84 ± 0.25	5.93 ± 0.31	4.05 ± 0.25	3.02 ± 0.13
Lungs				
Dose/mAs (mGy/mAs)	0.111 ± 0.005	0.030 ± 0.001	–	–
Dose/mAseff (mGy/mAseff)	0.113 ± 0.005	0.027 ± 0.001	–	–
Mean absorbed dose (mGy)	6.12 ± 0.27	4.58 ± 0.22	5.13 ± 0.23	3.66 ± 0.16

### 3.C Comparison with NCICT

The experimental acquisition parameters for each phantom and CT scanner were simulated with the software NCICT. TCM protocols were simulated with the batch module of the software.^17^

Percent differences between experimental measurements with TLDs and NCICT (eq. (4)) were within a 20% interval, with the highest value (19.3 ± 0.8%) corresponding to the pediatric thyroid dose measured with the Chest for Children protocol with 80 kV and TCM (Table 8). The lowest percent difference corresponds to the adult lung dose for the Ultra Low‐dose protocol [−(2.1 ± 0.1)%].

**Table 8:** Comparative evaluation between experimental and simulated organ doses for the adult and pediatric phantom

Adult lung doses						
Organ	Measurement	Chest protocols — constant tube current			Chest protocols — tube current modulation/TCM	
		Standard	Low‐dose	Ultra low‐dose	Auto mA	Auto + Smart mA
Lungs	TLD (mGy)	14.30 ± 0.72	3.88 ± 0.19	1.24 ± 0.06	11.90 ± 0.60	9.29 ± 0.46
	NCICT (mGy)	13.24	3.64	1.21	14.11	11.23
	Δ (%)	−(8.0 ± 0.9)	−(6.5 ± 0.3)	−(2.1 ± 0.1)	(15.7 ± 0.8)	(17.3 ± 0.9)%

Pediatric lung and thyroid doses					
Organ	Measurement	Chest protocols — constant tube current		Chest protocols — tube current modulation/TCM	
		80 kV	120 kV	80 kV	120 kV
Lungs	TLD (mGy)	4.58 ± 0.22	6.12 ± 0.27	3.66 ± 0.16	5.13 ± 0.23
	NCICT (mGy)	5.60	6.99	4.34	5.48
	Δ (%)	(18.2 ± 0.9)	(12.5 ± 0.6)	(15.6 ± 0.7)	(6.3 ± 0.3)
Thyroid	TLD (mGy)	5.93 ± 0.31	6.84 ± 0.25	3.02 ± 0.13	4.05 ± 0.25
	NCICT (mGy)	6.22	7.54	3.74	4.68
	Δ (%)	(4.6 ± 0.2)	(9.3 ± 0.3)	(19.3 ± 0.8)	(13.4 ± 0.8)

**Figure 3:**
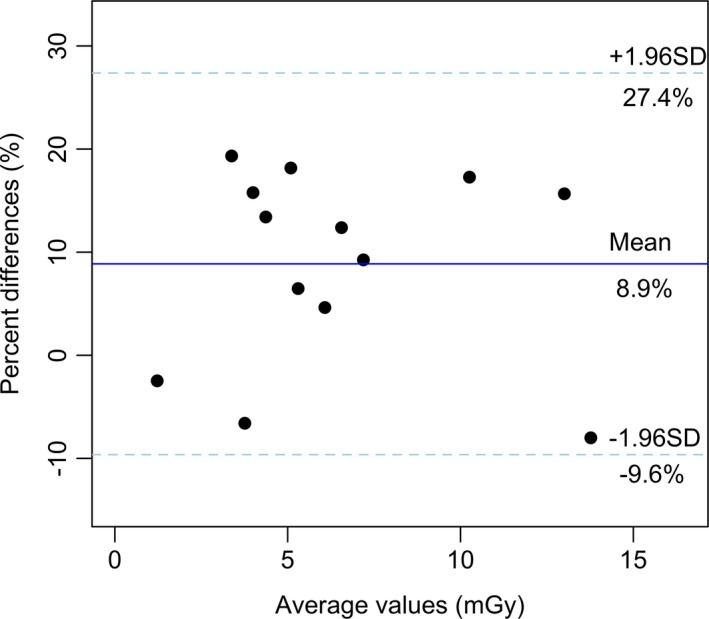
Bland–Altman plot of the percent differences against the mean of the organ doses obtained with the NCICT software and TLD measurements. The mean of the percent differences is presented in blue (8.9%) and the 95% limits of agreement are presented in the dashed lines.

The Bland–Altman plot^46^ is presented in Fig. 3. This picture presents the average of the percent differences between both methods (i.e., the bias) along with the 95% limit of agreement (dashed lines), which corresponds to the average dose $\left(\overset{¯}{D}=\frac{D_{\exp}+D_{\mathrm{sim}}}{2}\right)$ plus or minus 1.96 times the standard deviation $\left(\overset{¯}{D}\pm 1.96\times \text{SD}\right)$. This means that for any future sample, the differences between both methods should fall within this limit in about 95% of the trials. The upper limit of agreement is higher than the limit adopted in this study (20%): the highest difference found was (19.3 ± 0.8)% for the thyroid using 80 kV and TCM, which is in agreement with the 20% limit that has been adopted. Therefore, the results presented in both Table 8 and Fig. 3 demonstrate the compatibility between NCICT and the experimental method using TLD as proposed in this investigation within 20%.

## 4 DISCUSSION

This study proposes a methodology to determine lung‐absorbed doses in an adult anthropomorphic phantom, as well as lung and thyroid‐absorbed doses in a pediatric phantom, using thermoluminescent dosimeters in 10 different chest CT protocols. Results obtained with this method were compared within each other and with calculations performed with the NCICT software.

### 4.A SPR and chest protocols: adult phantom

#### 4.A.1 Scan projection radiograph

With the introduction of TCM systems, SPRs are being widely performed once they also serve as a reference of a patient's density and thickness for the TCM systems. Even though the imparted dose due to such irradiation is expected to be considerably smaller than for a CT procedure, it is relevant to estimate its value. The mean absorbed lung dose due to both AP and LAT SPR was estimated in 0.19 mGy. This estimation corresponds to about 1.3% of the dose absorbed by the lungs in STD protocol. This fraction increases to 4.9% and 15% when compared with LD and ULD protocols, respectively. For “Auto mA” and “Auto + Smart mA”, this contribution is 1.6% and 2.1%, respectively. Therefore, SPR contributes with relatively low doses to the lungs in STD protocols, but in protocols developed with the goal of reducing dose without losing image quality, its contribution may become significant.

Although SPR became an essential step prior to CT scans, there are not many published studies in the literature involving dosimetric aspects of SPR scans. Mini et al.^48^ investigated the dose absorbed by several organs due to one single protocol of SPR. The result for the dose absorbed by the lungs due to a chest SPR was 0.18 mGy. Even though Mini and colleagues do not specify in which projection SPR was taken and neither the scanner used, it was shown that the dose due to such procedure is relatively small, when compared with the other studied protocols. Moreover, the value reported is in good agreement with the value estimated in the present work.

#### 4.A.2 Chest CT with fixed tube current

Huda et al.^49^ proposed a methodology that considers Monte Carlo simulations and the CTDI_vol_ value reported by the scanner console to calculate organ‐absorbed doses for a 70 kg patient undergoing chest CT examination. According to proposed by Turner et al.,^50^ organ‐specific coefficients *f*_organ_ relating organ dose and CTDI_vol_ were determined for each tube voltage. For lung with 120 kV, *f*_lung_ = 1.50 ± 0.06. It was considered that this *f*_lung_ value is valid for any chest CT performed with tube voltage set to 120 kV for a 70 kg adult. Taking into account the CTDI_vol_ values from the protocols studied in the present work, the lung‐absorbed doses may be estimated using the methodology proposed by Huda et al.^49^ (Table 9). The results obtained are in good agreement with the measurements performed with TLDs.

**Table 9:** Lung‐absorbed doses due to the Standard, Low Dose, and Ultra Low‐Dose chest CT protocols estimated by the present work (with TLD measurements) and by the methodology proposed by Huda and Sandison.^49^ The relative difference was calculated as the percentage difference between the values estimated by both methodologies

	Standard	Low dose	Ultra low dose
Dose by TLD measurements (mGy)	14.3 ± 0.2	3.88 ± 0.19	1.24 ± 0.06
CTDI_vol_ (mGy)	10.03	2.76	0.92
Dose by Huda et al.^49^ (mGy)	15.0 ± 0.6	4.1 ± 0.2	1.38 ± 0.06
Relative difference (%)	4.7 ± 4.0	5.4 ± 4.8	10.1 ± 4.1

Finally, from Table 9 the comparison of adult protocols with fixed tube current is also extracted. These results show that lung doses could be reduced by 72.9 ± 0.8% when using the LD protocol, and by 91 ± 1% when using the ULD protocol, in comparison to the STD protocol.

#### 4.A.3 Chest CT with TCM

TCM systems provide dose reduction by adapting the tube current according to patient anatomy and attenuation properties. For both TCM protocols, the tube current was set to rely between 80 and 300 mA, tube current‐time product being 48–180 mAs. For reference, the equipment used double SPR (LAT and AP).

The lung dose reduction achieved with “Auto mA” mode was 16.8 ± 1.2% compared to the STD protocol. Similarly, 35.0 ± 2.5% reduction was achieved with the “Auto + Smart mA” mode. Similar dose reduction levels for TCM are reported in literature.^18^, ^51^

### 4.B Chest protocols: pediatric phantom

#### 4.B.1 Fixed tube current

Dose estimates for thyroid and lungs were comparable when the phantom was irradiated with 80 kV and with 120 kV with fixed tube current. Decreasing the tube voltage from 120 to 80 kV while increasing the tube current‐time product from 55 to 146 mAs reduces both thyroid dose by 13.3 ± 0.8% (from 6.84 to 5.93 mGy) and lung dose by 25.2 ± 1.6% (from 6.12 to 4.58 mGy) (Table 10).

**Table 10:** Comparison among absorbed doses for thyroid and lungs when using fixed mA with 120 and 80 kV

	Dose (mGy)		
	120 kV, 55 mAs	80 kV, 146 mAs	Percent decrease
Thyroid	6.84 ± 0.25	5.93 ± 0.31	13.3 ± 0.8%
Lungs	6.12 ± 0.27	4.58 ± 0.22	25.2 ± 1.6%

Dose values estimated for the lungs and thyroid are similar, since these organs were irradiated by the primary beam of the CT scanner. Considering the relative quantity $\frac{\text{organ}\;\text{dose}}{{\text{mAs}}_{\mathrm{eff}}}$ reported in Table 7, in the Chest for children protocol with 120 kV, the thyroid dose per effective mAs was 0.115 mGy/mAs_eff_ for the 5‐yr‐old phantom. The lung dose per effective mAs was 0.113 mGy/mAs_eff_. Decreasing the tube voltage to 80 kV reduced the thyroid dose to 0.037 mGy/mAs_eff_ and the lung dose to 0.027 mGy/mAs_eff_.

A similar behavior was reported in the study conducted by Fujii et al.^52^ The authors performed organ doses measurements in a 1‐yr‐old pediatric phantom (ATOM Model 704‐C, CIRS, Inc., Norfolk, VA, USA) due to a 120 kV chest CT protocols. According to those authors, dose values for lungs and thyroid were comparable, indicating that the thyroid was irradiated by the primary beam as well as the lungs. In that study the thyroid dose per effective mAs was 0.234 mGy/mAs_eff_ and the lung dose per effective mAs was 0.238 mGy/mAs_eff_.

In the study conducted by Mathews et al.,^53^ the authors evaluated the cancer risk in pediatric patients after their exposure to ionizing radiation from CT examinations. The cohort had examinations performed from 1985 to 2005 and, overall, cancer incidence was 24% higher for exposed people than for unexposed people. In particular, an increased incidence rate ratio (IRR) was reported for several types of cancer (e.g., digestive organs, melanoma, brain), including thyroid. The authors argue that even though modern CT scanners are likely to yield to lower radiation doses, it is essential to limit CT examinations to cases that present a clear clinical indication, particularly for pediatric patients.

#### 4.B.2 TCM protocols

Table 11 shows the comparison of the absorbed organ doses when TCM modulation was turned on for both tube voltages (80 and 120 kV).

**Table 11:** The absorbed doses for thyroid and lungs when using fixed mA and TCM with 120 and 80 kV

	120 kV, 55 mAs	120 kV, 30–54 mAs (TCM)	Percent decrease (%)
Thyroid	6.84 ± 0.25	4.05 ± 0.25	40.8 ± 2.9
Lungs	6.12 ± 0.27	5.13 ± 0.23	16.2 ± 1.0

	80 kV, 146 mAs	80 kV, 77–145 mAs (TCM)	Percent decrease (%)
Thyroid	5.93 ± 0.31	3.02 ± 0.13	49.1 ± 3.3
Lungs	4.58 ± 0.22	3.66 ± 0.16	20.1 ± 1.3

	120 kV, 30–54 mAs (TCM)	80 kV, 77–145 mAs (TCM)	Percent decrease (%)
Thyroid	4.05 ± 0.25	3.02 ± 0.13	25.4 ± 1.9
Lungs	5.13 ± 0.23	3.66 ± 0.16	28.7 ± 1.8

According to the results in Table 11, TCM can reduce the organ doses by 49.1 ± 3.3% in the pediatric phantom when setting a tube voltage of 80 kV and by 40.8 ± 2.9% when using 120 kV. In the clinical practice extracted from the data collected, the majority (>95%) of examinations were performed with 120 kV and TCM, while a few examinations were performed with 80 kV and TCM. From Table 11, switching the kV from 120 to 80 keeping the TCM in both cases would save up to 25.4 ± 1.9% of thyroid doses and up to 28.7 ± 1.8% of lung doses, maintaining the necessary image quality for diagnostic purpose. Therefore, a possibility of optimization was identified, which is in progress of implementation and validation.

In particular, it is essential to evaluate the image quality when aiming at protocol optimization. There are several studies reporting different tools to assess clinical image quality,^54^, ^55^, ^56^, ^57^ although on the other hand there are several studies showing that a radiologist tend to select images in which a given objective parameter (e.g., contrast resolution) is higher.^54^ In the study proposed by Rehani^54^ the author presents several arguments supporting the subjective image quality evaluation by a radiologist. In this sense, the images acquired for a number of patients performing routine chest CT at 80 and 120 kV were evaluated by a radiologist from InRad in the present investigation. All important structures were visible in both examinations, thus indicating that 80 kV with TCM might be adequate when performing routine chest examinations in children within this age range.

The overall reduction in absorbed organ dose with TCM adjustments is in good agreement with the literature, although TCM differs per CT scanner and protocol. Coursey et al.^58^ obtained a mean absorbed dose reduction of 53% for the lungs and 56% for the thyroid, when using TCM in the z‐direction for the same reference phantom. Alibek et al.^59^ reports 32% of dose reduction for chest pediatric CT examinations when using TCM. *In vivo* studies^19^ in CT radiation dose show an average body dose reduction of 11% in pediatric patients with similar anatomy as the phantom used in this study.

However, some studies report small increases in absorbed organ dose in pediatric subjects due to TCM.^18^ In the study conducted by Karmazyn and colleagues,^19^ the authors discourage the use of TCM in very small pediatric patients due to the uniformity of their body shape to preclude the possibility of an unnecessary high current‐time product. Therefore, the dose reduction strategy must always be discussed between clinical and physics staff, especially for pediatric patients.

**Figure 4:**
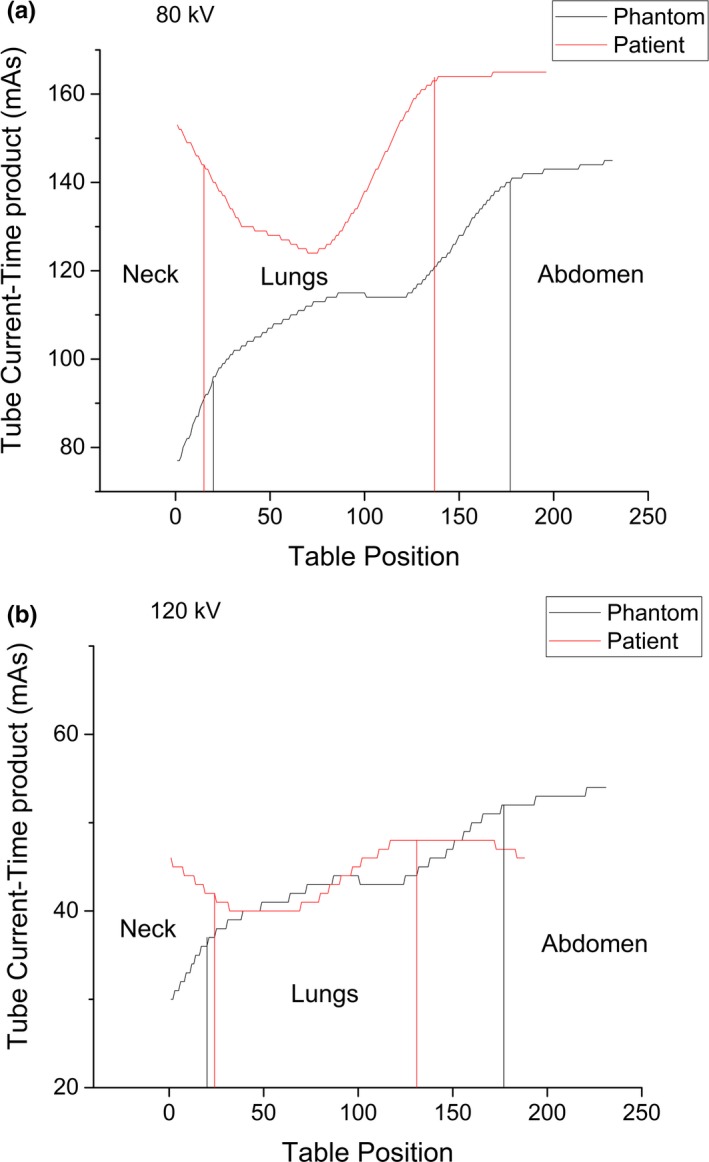
Variation of the tube current‐time product over the longitudinal axis of the patient and the phantom, from the neck (Table position 0) to the abdomen with a tube voltage of 80 and 120 kV. The tube current‐time product decreases in the direction of the abdomen of the phantom and increases in the direction of the abdomen of the patient. In both cases, the area within the black vertical lines corresponds to the position of the lungs inside the phantom, and within the red vertical lines to the position of the lungs inside the patient.

Due to differences in anatomy (e.g., acquisition with arms elevation) the tube current‐time product over the longitudinal direction might be higher in patients than in phantoms at the thyroid level, since TCM tries to compensate the difference by increasing the tube current‐time product. Figure 4 presents the comparison of the tube current as a function of the table position among the Chest for children protocol applied in the phantom and in a patient with 80 and 120 kV. In both situations, the tube current‐time product is higher for the patient than for the phantom in the region around the neck (table position 0). For the patients, the value is decreasing in the direction of the lungs. For the phantom, the tube current‐time product starts lower in the neck and increases in the direction of the lungs. Outside the lungs, this value presents a similar trend for patients and for the phantom with both tube voltages.

### 4.C Comparative evaluation with NCICT

Experimental and simulated results were in agreement within 20%. Small differences are mainly related to anatomical difference between the computational human phantoms built in NCICT and the physical phantoms used for dose measurements. Despite such differences, the experimental methodology presented in this study showed to be adequate for dose evaluation.

Monte Carlo simulations to estimate organ‐absorbed doses have become a common subject. In the study conducted by Huang and colleagues, for instance, the authors evaluated the effect of organ based TCM on the reduction in eye lenses dose using Monte Carlo simulation.^60^ Fujii et al.,^52^ for instance, compared the experimental results obtained with silver‐activated phosphor glass dosimeter with results simulated by ImpactMC (CT Imaging GmbH, Germany) for an adult physical phantom and a 1‐yr‐old physical phantom. Percent differences reported by these authors are within 13% for organs that were within the scan range and the authors considered measured and simulated results to be in good agreement. In the study conducted by Dabin et al.,^44^ the authors performed organ dose measurements in a 5‐yr‐old anthropomorphic phantom for five different CT scanners from four manufacturers. The authors measured absorbed doses to 22 organs by directly applying TLDs inside the organs of the phantom for head‐to‐torso acquisitions. These values were compared to calculations performed with the software NCICT and two main results of this study can be highlighted. First, for most organs the difference between measured and simulated absorbed doses was within 20%, similar to results found in this study. In addition, the authors developed a voxelized phantom based on the CIRS ATOM phantom used for the experimental measurements and performed the simulations using this voxelized phantom. Percent differences in this case were within 10.4%. This result confirms that the main cause of differences between simulations and experiments are associated to discrepancies in simulated and measured phantoms anatomies. The length of the necks of the voxelized phantom and the physical phantom are particularly different from each other, which explains the highest percent differences among thyroid doses obtained experimentally and with NCICT (Table 7).

As previously described (Section Purpose), one of the limitations associated with Monte Carlo simulations of CT scanners is the need of confidential technical parameters, which are not always measurable. In particular, the NCICT code is entirely based on a reference CT scanner and relies on the fact that CTDI_vol_‐normalized organ doses do not depend on the scanner. Although this independency can indeed provide fair dose estimations, NCICT is intrinsically limited to the technical parameters of the reference CT scanner. Therefore, when accurate dose values are required, measurements using TLD and physical anthropomorphic phantoms are more reliable. In particular, besides providing a high spatial resolution because of their small sizes, Lithium Fluoride TLD dosimeters used in these experiments are tissue‐equivalent^16^ and they were calibrated using the same CT scanner used for the measurements (thus the same X ray beam), therefore it was not necessary to correct for the energy dependence.

### 4.D Clinical benefits and limitations

The clinical motivation for this study was the general evaluation of the practices related to CT procedures performed in a clinical institution. The experimental measurements were performed in order to have a *more reliable estimate* of the organ doses in such procedures. A limitation of this study refers to the use of two sizes of anthropomorphic phantoms and two organs only. However, this is an accurate method that can be applied in a wide range of phantoms and even in *post‐mortem* subjects according to a given clinical need by other authors. Additionally, because inherent limitations of Monte Carlo simulations, experimental measurements with TLDs offer more accurate results.

The main challenge related to the clinical translation is due to the image quality of optimized protocols, which needs to be carefully addressed before implementing any kind of adjustment to the clinical routine.

## 5 CONCLUSIONS

An experimental approach was applied in this study to evaluate organ doses in anthropomorphic phantoms in different chest CT protocols. This methodology has proven to be efficient for measurements of doses to organs within the scan regions but its applicability to different situations must be evaluated, especially when the organ is not directly irradiated by the primary CT beam. Nonetheless, because of the limitations associated with Monte Carlo simulations, experimental measurements with TLDs should be the approach of choice when more accurate dose values are required. Finally, findings of the present investigation may pave the way to decrease radiation dose whereas the image quality could be potentially preserved with the use of first generation and model based iterative reconstruction methods. In particular, dose reduction in up to 28.7% on the absorbed dose was reported for pediatric protocols with a change from 120 to 80 kV using TCM; TCM and ultra low‐dose adult protocols can lead up to 35.0% and 90.0% in dose reduction, respectively, when compared with the standard adult protocol, which is performed with fixed mAs. Further investigations considering other radiosensitive organs and other protocols must be conducted as a step toward the implementation of optimization strategies.

## CONFLICT OF INTEREST

The authors declare that they have no conflict of interest.

## Figures and Tables

**Figure Fig. A1:**
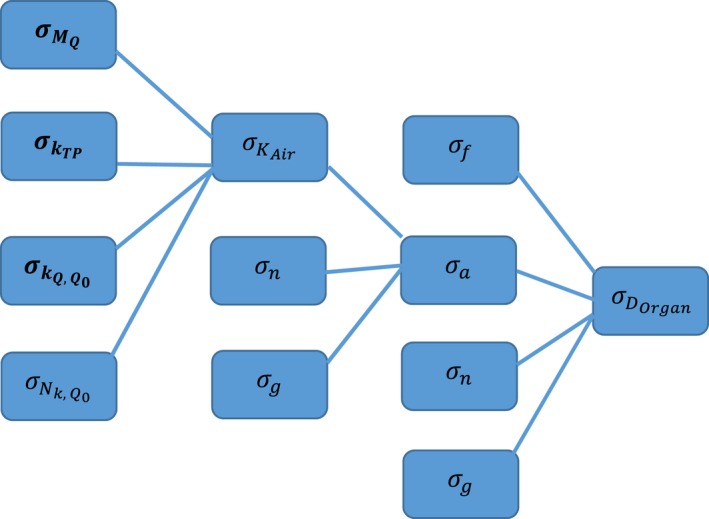
Scheme illustrating all the uncertainties used to the overall uncertainty for lung‐absorbed dose estimation.

## ENDNOTE

1 NCICT program can be obtained from National Cancer Institute by contacting Dr. Choonsik Lee at http://ncidose.cancer.gov.

## APPENDIX A UNCERTAINTIES ESTIMATION

### A.1

The uncertainties considered to calculate the overall uncertainties of lung‐ and thyroid‐absorbed dose estimates are summarized in Fig. A1. $\sigma_{M_{Q}}$is the uncertainty in the ionization chamber reading (in Coulombs) for a X ray beam quality Q, $\sigma_{N_{k,Q_{0}}}$ is the uncertainty of the calibration coefficient given by the IC calibration report, $\sigma_{k_{Q,Q_{0}}}$ refers to the correction factor for a radiation beam quality Q regarding the ionization chamber's calibration beam quality Q_0_, $\sigma_{k_{\mathrm{TP}}}$ is due to the correction factor for temperature and pressure, $\sigma_{k_{\mathrm{Air}}}$ is the composed uncertainty for air kerma values, $\sigma_{n}$ is the uncertainty of the TL values from the different TLDs inside a measuring group, $\sigma_{g}$ is the systematic uncertainty regarding the TLD group selection (i.e., 6.5%),^25^ $\sigma_{a}$ refers to the calibration curve of TL values and air kerma, $\sigma_{f}$ corresponds to uncertainties on the organ mass fraction inside each physical slice of the phantoms,^32^ and finally $\sigma_{D_{\mathrm{Organ}}}$ is the overall uncertainty to the organ dose estimates obtained with the propagation of all components. Confidence level considered is 68.3%. (*k* = 1).
